# Exceptional response to neoadjuvant targeted therapy with the selective RET inhibitor selpercatinib in *RET*-fusion-associated sarcoma

**DOI:** 10.1007/s00432-022-04496-y

**Published:** 2022-12-05

**Authors:** Karin G. Schrenk, Wolfram Weschenfelder, Christian Spiegel, Abbas Agaimy, Robert Stöhr, Arndt Hartmann, Nikolaus Gaßler, Robert Drescher, Martin Freesmeyer, Amer Malouhi, Florian Bürckenmeyer, René Aschenbach, Ulf Teichgräber, Christine Kögler, Matthias Vogt, Gunther O. Hofmann, Andreas Hochhaus

**Affiliations:** 1 Department of Hematology and Internal Oncology, Clinic of Internal Medicine II, University Hospital Jena, Am Klinikum 1, 07747 Jena, Germany; 2 Clinic of Trauma-, Hand- and Reconstructive Surgery, University Hospital Jena, Jena, Germany; 3 Institute of Pathology, Comprehensive Cancer Center Erlangen-EMN (CCC ER-EMN), University Hospital Erlangen, Friedrich-Alexander University Erlangen-Nürnberg, Erlangen, Germany; 4 Institute of Forensic Medicine, Section of Pathology, University Hospital Jena, Jena, Germany; 5 Clinic of Nuclear Medicine, University Hospital Jena, Jena, Germany; 6 Institute of Diagnostic and Interventional Radiology, University Hospital Jena, Jena, Germany; 7 Clinic of General- and Visceral Surgery, Malteser Hospital St. Marien, Erlangen, Germany; 8 Clinic in the Medicenter PartGmbB, Erlangen, Germany

**Keywords:** *RET*-fusion, Sarcoma, Selpercatinib, Targeted therapy, TRIM33

## Abstract

With the increasing use of next-generation sequencing, highly effective targeted therapies have been emerging as treatment options for several cancer types. Recurrent gene-fusions have been recognized in sarcomas; however, options for targeted therapy remain scarce. Here, we describe a case of a sarcoma, associated with a *RET::TRIM33*-fusion gene with an exceptional response to a neoadjuvant therapy with the selective RET inhibitor selpercatinib. Resected tumor revealed subtotal histopathologic response. This is the first report of successful targeted therapy with selpercatinib in *RET*-fusion-associated sarcomas. As new targeted therapies are under development, similar treatment options may become available for sarcoma patients.

Gene-fusions are characteristic of a variety of sarcomas and are considered major driver mutations. Prominent examples are *EWSR1::FLI1*-fusion in Ewing sarcoma, *SS18::SSX1/2/4*-fusion in synovial sarcoma and *NTRK*-fusions in infantile fibrosarcoma and other rare neoplasms. Detection of specific gene-fusions is used for histopathological diagnosis, but targeted therapy options are not available for most of these gene-fusions. Exceptions are tumors driven by receptor tyrosine kinase-fusions including *NTRK*, *ALK*, *ROS1* and *RET*. In rare cases with *NTRK*-fusions, targeted therapy has been shown to be highly effective. The use of larotrectinib in *NTRK*-fusion-positive cancers had a 75% response rate and an ongoing response of 71% in metastasized or irresectable *NTRK*-fusion-positive cancers after one year of treatment (Drilon et al. 2018). With the increasing use of new technologies, like next-generation sequencing and RNA sequencing, new fusions are detected in sarcomas. Recently, rearranged during transfection (*RET*)-fusion-associated sarcomas have been reported in a series of six cases (Antonescu et al. 2019). All patients had *RET*-fusions with an identical break point in exon 12, which retains the tyrosine kinase domain in the fusion oncoprotein. Two patients with a malignant histology had an aggressive course with distant metastasis. We report an highly effective neoadjuvant and adjuvant treatment of a patient with a *RET::TRIM33*-fusion sarcoma with the selective RET inhibitor selpercatinib.

A 33-year-old male patient presented with a mass within his left triceps brachii muscle, progressive in size over a period of four months. MR imaging revealed a 6.3 × 4.6 × 7.3 cm tumor suspicious of sarcoma (Fig. 1A, B, red arrows). ^18^F-FDG-PET/CT scan demonstrated intensive glucose hypermetabolism of the lesion (Fig. 1C). No metastases were detected. Biopsy revealed a spindle cell mesenchymal neoplasm, suspicious of fusion-associated unclassified sarcoma (Fig. 2B). Based on our routine diagnostic strategies to submit any unusual or unclassified mesenchymal neoplasm with monomorphic morphology (excluding undifferentiated pleomorphic sarcoma) for targeted RNA sequencing, molecular analysis was performed. Targeted RNA sequencing showed a *RET::TRIM33*-fusion (Fig. 2A). In this fusion, exons 1–10 of the *TRIM33* gene were fused with exons 12–20 of the *RET* gene including the ring-box-coiled-coil unit of TRIM33 and the protein tyrosine kinase domain of RET. After approval by our molecular tumor board and the sarcoma board, the patient commenced selpercatinib 160 mg twice daily per os. Potential side effects of selpercatinib such as arterial hypertension, liver enzyme elevation, hyponatremia, elongation of QT-time and hemorrhages were monitored by daily blood pressure self-measurements and weekly medical surveillance including electrocardiogram and control of electrolytes. No side effects were detected. Starting at day 3 of the targeted therapy, the patient noticed an obvious shrinkage of the tumor. After 12 days, the tumor had almost completely disappeared on clinical examination. ^18^F-FDG-PET/CT scan revealed metabolic reduction of 56%, representing a partial response according to PERCIST 1.0 criteria and volume reduction of 45% compared to the initial finding (Fig. 1F). Because of the impressive regression of the tumor, selpercatinib treatment was continued for another 2 weeks. MR imaging demonstrated a further reduction of tumor volume to 4.6 × 1.0 × 4.6 cm (Fig. 1D, E). Due to the change of − 37% of the largest tumor diameter, response evaluation according to RECIST 1.1 is a partial response. However, up to this time, tumor volume had decreased by 90%. On MR-angiography, hypervascularity of the tumor was demonstrated and embolization of the tumor-supplying artery prior to surgery was performed. The sarcoma was resected with wide margins. Histology demonstrated near-total pathological response (ypT1, ypNX, L0, V0, Pn0) with dominant regressive, fibrosclerotic manifestation and minimal (5–10%) vital residual tumor cells. The resection margins were free of tumor (Fig. 2C). Locoregional irradiation with 56 Gy and boost to 65 Gy was applied to the local resection site. Adjuvant selpercatinib at constant dose of 160 mg twice daily per os was continued over a period of 4 weeks.

**Fig. 1:**
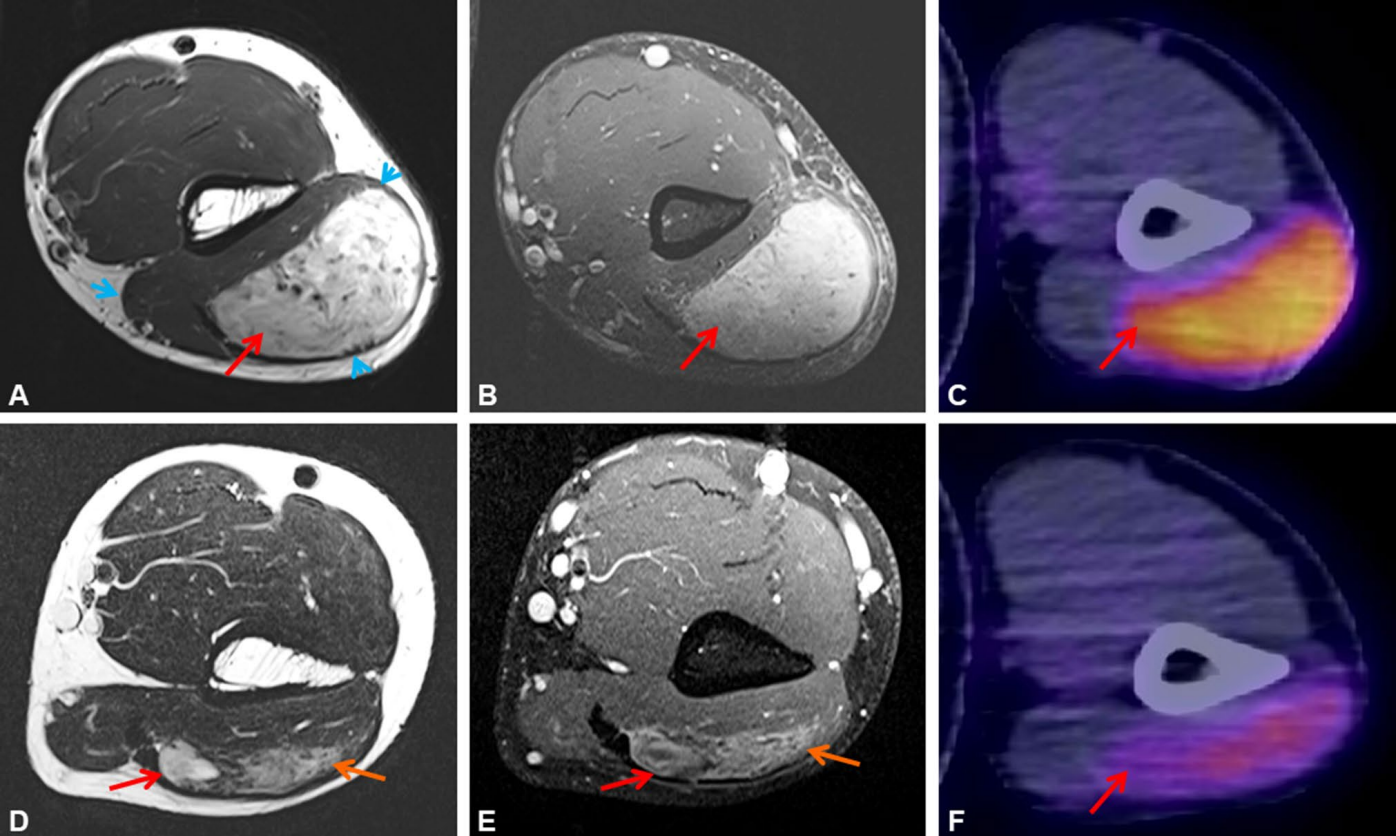
Well-defined mass (red arrow) within the left triceps brachii muscle (blue arrowheads). The mass is hyperintense on T2-weighted MR imaging (**A**) and shows strong contrast enhancement (T1-weighted image) (**B**). MR imaging performed 28 days after treatment initiation. Further shrinkage of the tumor, with solid (red arrow) and diffuse (orange arrow) residual areas (**D**). Remaining mild, inhomogeneous contrast enhancement (red  and orange arrow) (**E**). ^18^F-FDG-PET/CT on initial presentation reveals glucose hypermetabolism of the lesion (SUV_max_ 6.6) (**C**). ^18^F-FDG-PET/CT performed 12 days after treatment initiation with selpercatinib. Markedly reduced glucose hypermetabolism of the tumor (red arrow; SUV_max_ 2.9, SUV_max_ of the triceps brachii muscle 0.9) (**F**)

**Fig. 2:**
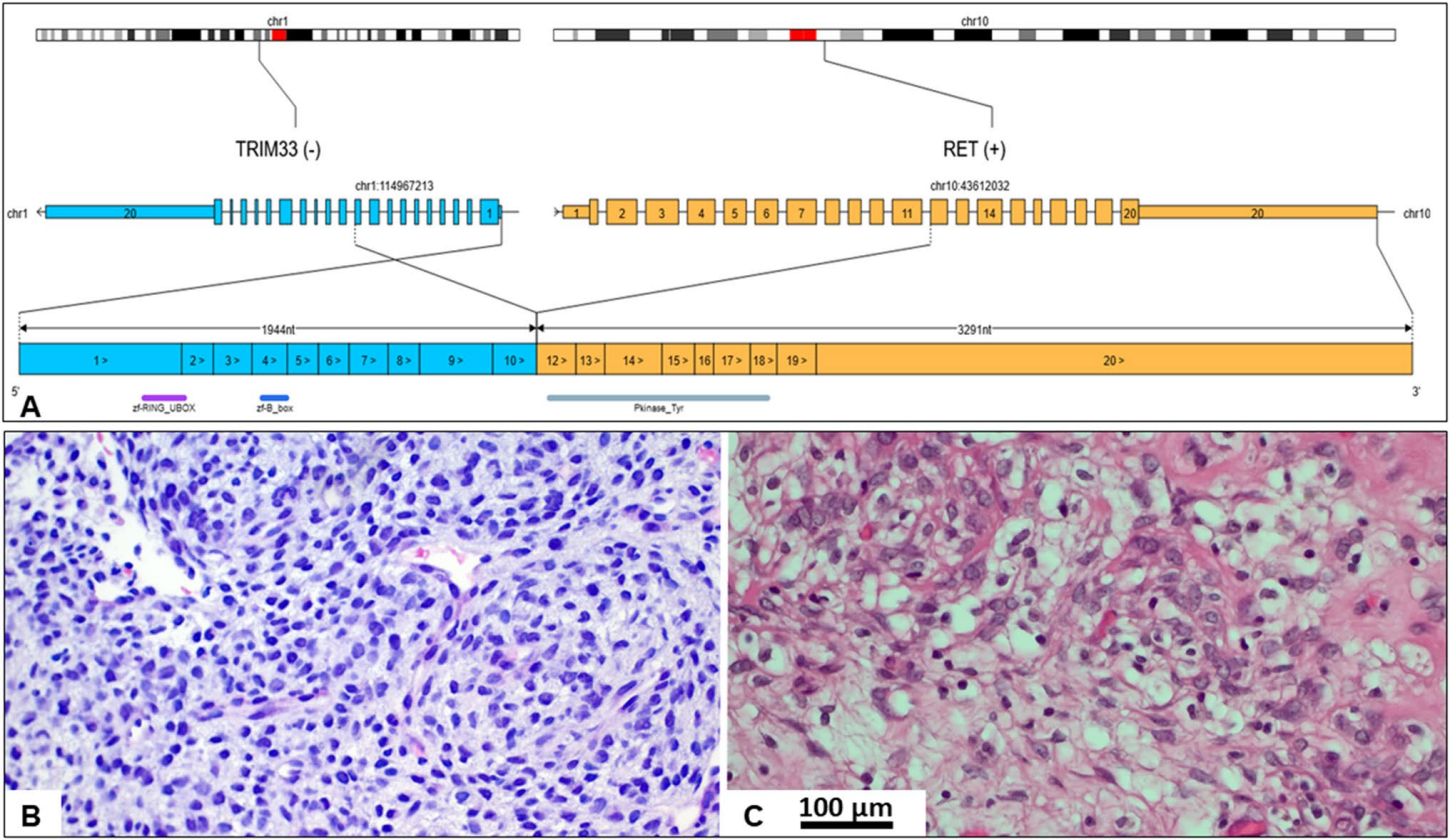
Graphical overview of the detected *TRIM33::RET*-fusion. Exons 1–10 of the *TRIM33* gene were fused with exons 12–20 of the *RET* gene including the protein tyrosine kinase domain of RET (image was created using the public Oviz-Bio platform (https://bio.oviz.org/demo-project/analyses/FusionTransJunc) (**A**). Biopsy showed a highly cellular neoplasm with monomorphic spindled and ovoid cells (400 × magnification) (**B**). After 4 weeks of treatment with selpercatinib, extensive fibrosclerotic regressive changes with thrombotic, sclerotic blood vessels and a minimal vital cell count of 5–10% was demonstrated in the resected triceps brachii muscle (400× magnification) (**C**)

Tripartite motif-containing 33 or transcriptional intermediary factor 1γ (TRIM33, TIF1γ) protein belongs to the RING type E3 ubiquitin ligase family and regulates TGFß/Smad signaling. It functions either as tumor suppressor or promotor in different cancers (Yu et al. 2019). The *RET* gene encodes a transmembrane receptor that functions as tyrosine kinase protein. Binding of ligands to the receptor induces receptor dimerization and activation of downstream signaling pathways involved in cell growth, differentiation and development of the nervous system (Adashek et al. 2021; Subbiah et al. 2020). *RET*-fusions have been identified in several cancer types including 2% of lung carcinomas, 10–20% of thyroid carcinomas and in low frequency in other tumors (Thein et al. 2021). Selpercatinib was highly effective in lung and thyroid cancer treatment (Drilon et al. 2020; Wirth et al. 2020). In lung cancer, the objective response rate (ORR) in second-line treatment after cisplatin-containing therapy was 69% and the median duration of response was 17.5 months. In the first-line treatment, ORR was reported to be 85%. 90% of the responses were ongoing at six months (Drilon et al. 2020). An update of the LIBRETTO-001-study with a longer follow-up, and a higher patient number confirmed a sustained efficacy of selpercatinib in patients with non-small cell lung cancer (Drilon et al. 2022). In *RET*-mutant medullary thyroid cancer and *RET*-fusion-positive thyroid cancers with different fusion partners, similar response rates were observed (Wirth et al. 2020).

Because of a locally advanced T2 tumor in our patient with high risk of metastasis, selpercatinib was used as both neoadjuvant and adjuvant treatment. The short-term neoadjuvant therapy was highly efficient since the clinical response was visible after 3 days of treatment. After 12 days, a remarkable reduction in tumor volume and metabolic activity could be demonstrated. After 4 weeks of treatment, a further impressive tumor reduction was observed. Compared to the primary tumor volume, the final reduction in tumor size was 90%. The sarcoma was successfully resected.

To the best of our knowledge, this is the first report of an effective therapy with the selective RET inhibitor selpercatinib in neoadjuvant treatment of a *RET*-fusion-associated sarcoma. This case emphasizes the need to intensify the search for future targeted therapies in fusion-associated sarcomas.

## Data Availability

Data is available
